# Natural Silicated Mineral Springs of New England

**Published:** 1914-12

**Authors:** Felix Von Oefele

**Affiliations:** New York. 326 E. 58th St.


### Natural Silicated Mineral Springs of New England.

DR. FELIX VON OEFELE, New York

A second group of low mineralized springs of New England contain a relatively high amount of silicon. These springs are not located in straight lines as the nitrated springs are. These silicated springs contain very little or no nitric acid or nitrates. As stated above they are rich in silicic acid and irregularly distributed. For the nitrated springs it was stated that the dissolved minerals originated from the magma. The minerals of the silicated springs are evidently dissolved from more superficial beds.

There are many groups of silicated springs in the state of Maine. The geological conditions are these: Cambrian and lower Ordovician beds. These springs are Glenwood Mineral Spring (11.76—0.0094), Mount Hartford Cold Spring (19.50—0.0046), Highland Spring, Me., (21.78—0.0052), Keystone Mineral Spring (11.16—0.0104), Pine Spring (22.51—0.0033). The first figure, the %of silicic acid calculated to total solids, the second figure means % of total solids.

A second group of silicated springs is found on both sides of the eastern boundary between New Hampshire and Massachusetts in palaeozoic and old volcanic strata. Lafayette Mineral Springs, N. H., (14.38—0.0142), Londonderry Spring, N. H., (20.48—0.0069), Amherst Mineral Spring, N. H., (25.30—0.0088), Ballardvale Spring, Mass., (34.2—0.0021), Pack Monadnock Spring, N. H., (34.0—0.0041). This group contains the highest percentage of silicic acid. If we would draw a straight line through Pack Monadnock and Ballardvale Springs in some western direction we would reach Equinox Spring Vermont, (45.09—0.0078). But this spring was mentioned in the natural nitrated mineral waters as a starting spring of the second small, north to south fault of New England nitrated waters. The relative low amount of nitric acid and the very high amount of silicates, would preferably place this in the second silicated group.

A third group, arranged nearly fault like, is situated in the State of Rhode Island. Nobscot Mountain Spring, Mass., (20.21—0.0053), Holly Mineral Spring, R. I., (17.45—0.0067), Ochee Mineral Spring (13.76—0.0113). In some way Stafford Spring, Tolland Co., Conn., (28—0.0121) and Stock Mineral Spring, Conn., (25—0.0057) may be annexed to this group.

A fourth group is situated in the south western part of Connecticut. Live Oak Spring (15.30—0.174), Arethusa Spring (24.85—0.0041), Mohican Spring (23.32—0.0054).

The therapeutic importance of silicon is very high. We cannot now speak extensively of the silicate thermal waters, because all the mentioned New England waters are cold, but in Germany, Baden-Baden contains nearly 5% of total solids as Silicic acid. The different Springs of Arkansas Hot Springs contain, almost between 15 and 20% of total solids silicic acid. The percentage of silicic acid in cold European Springs is mostly low; remarkably so is Julianen Brunen, and Georgen Brunnen at the little spring town, Eilsen, near Bueckeburg Germany, which contain one to two per cent. of total solids silicic acid. I have found from the data at hand, from the analysis all the other European Mineral Springs contain less silicic acid.

The Silicic Acid is very important in the metabolism of the foetus and in every tissue of the later life that keeps in the foetal condition. The organism becomes poorer on Silicic Acid in the average percentage in the higher age. The metabolism of premature age is partly a premature leakage of Silicic Acid. On account of the low amount of Silicic Acid in the European Springs, as yet silicated springs have not been sufficiently studied, but in New England in the neighborhood of these springs, people live to an old age. These springs contain a higher amount of Silicic Acid than the mentioned German Springs resort, Eilsen. It seems therefore to the writer, that it is the duty of the American physician to use some of the mentioned places of New England containing springs, for their aged people as summer resorts, especially for old people in danger of arterioscerosis or atheromatosis.

In every tissue, any excessive pathologic deposit of lime, is also an indication for silicates. Through the entire zoologic kingdom a deposit of Silicic Acid excludes a deposit of lime and vice versa. If Europe was as rich in silicated waters as New England is, the value of silicated waters would be more thoroughly studied and become better known.

326 E. 58th St.

---

**Laryngo-Bronchoscopy Statistics.** Daniel W. Layman, Indianapolis, "Jour. of Ind. State Med. Asso.," September, 1914.

| | Man. | Woman. | Child. | Infant. |
|---|---|---|---|---|
| Diameters | mm. | mm. | mm. | mm. |
| Trachea ............... | 15-22 | 13-18 | 8-11 | 6-7 |
| Glottis ................ | 12-15 | 10-13 | 8-10 | 5-6½ |
| Right main bronchus ... | 12-16 | 10-15 | 7- 9 | 5-6 |
| Left main bronchus .... | 10-14 | 9-13 | 6- 8 | 5-6 |
| Tubular spatulas (Shoonmaker's) ...... | 14 | 12 | 10 | 7 |
| Extension tubes ........ | 11 | 9½ | 7½ | 6½ |
| Tubular spatulas (Brunings') ......... | 12 | 10 | 8½ | 7 |

The Brunings instruments are proportionately smaller.

